# Magnetic Particle Imaging tracks the long-term fate of *in vivo* neural cell implants with high image contrast

**DOI:** 10.1038/srep14055

**Published:** 2015-09-11

**Authors:** Bo Zheng, Tandis Vazin, Patrick W. Goodwill, Anthony Conway, Aradhana Verma, Emine Ulku Saritas, David Schaffer, Steven M. Conolly

**Affiliations:** 1 Department of Bioengineering, University of California at Berkeley, Berkeley, CA 94720, USA; 2 Department of Chemical and Biomolecular Engineering, University of California at Berkeley, Berkeley, CA 94720, USA; 3 Department of Molecular and Cell Biology, University of California at Berkeley, Berkeley, CA 94720, USA; 4 Magnetic Insight, Inc., Newark, CA 94560, USA; 5 Department of Electrical Engineering and Computer Science, University of California at Berkeley, Berkeley, CA 94720, USA

## Abstract

We demonstrate that Magnetic Particle Imaging (MPI) enables monitoring of cellular grafts with high contrast, sensitivity, and quantitativeness. MPI directly detects the intense magnetization of iron-oxide tracers using low-frequency magnetic fields. MPI is safe, noninvasive and offers superb sensitivity, with great promise for clinical translation and quantitative single-cell tracking. Here we report the first MPI cell tracking study, showing 200-cell detection *in vitro* and *in vivo* monitoring of human neural graft clearance over 87 days in rat brain.

The evaluation of cell transplantation efficacy and integration currently relies on destructive analytical methods like histology or functional improvements that can take months to manifest^1^. Inaccuracies in cell localization during initial delivery are common, with one clinical study reporting a 50% misadministration rate by experienced operators under ultrasound guidance^2^. Late detection of such outcomes can seriously impact the development of cell-based therapies. Clinical translation and adoption of cell therapies could be accelerated by safe, quantitative *in vivo* imaging methods^3^.

All standard preclinical imaging modalities have been used to track cells *in vivo* with varying degrees of success^3^. Bioluminescence and fluorescence imaging are the most common preclinical cell-tracking techniques; however, shallow optical penetration depth prevents linear quantification and clinical translation. Nuclear imaging techniques like Positron Emission Tomography (PET) have excellent tracer sensitivity and depth penetration, but face limitations in tracer half-life and radiation dose.

Clinically, most cell tracking studies have used super-paramagnetic iron oxide (SPIO) labeled cells, because SPIO-based methods have minimal effects on cell viability, proliferation, and differentiation^4^,^5^,^6^,^7^, along with excellent depth penetration and *in vivo* persistence measured in months. However, the primary challenge for MRI-based SPIO cell tracking is that SPIOs induce MRI signal dropouts that are difficult to distinguish from tissues with naturally low MRI signal (e.g., bones, tendon, lungs, or any tissues near air). Moreover, MRI methods with positive contrast suffer from robustness and sensitivity challenges^8^,^9^. Until now, no technique has been able to combine high specificity with sensitive and quantitative imaging of the distribution and *in vivo* fate of SPIO-labeled cells^3^,^4^.

Magnetic Particle Imaging^10^,^11^ is a technique introduced in 2005 by Philips Research that directly images the intense magnetization of SPIOs rather than indirectly detecting SPIOs via MRI signal dropouts. Briefly, MPI uses a magnetic gradient field to saturate all SPIO magnetization outside a central field-free region, which we show as a “field-free line”, or FFL, in Fig. 1a. To produce an image, the FFL is rapidly rastered over an imaging volume via the application of a time varying magnetic field (called a “drive field”) produced by an electromagnet. As the FFL traverses a location containing SPIO nanoparticles, the ensemble SPIO magnetization responds by changing in magnitude and orientation^12^. This time-varying particle magnetization induces a voltage in the receiver coil, which can be assigned to the instantaneous FFL location to produce a MPI image^11^,^13^. Importantly, while the magnetization behavior of superparamagnets is nonlinear, the voltage signals generated in MPI are linearly proportional to the number of SPIOs at the instantaneous FFL location, enabling quantification of SPIO number^13^. The MPI induction signal is detectable with even a miniscule mass of tracer (5 ng Fe/voxel in our projection MPI scanner) because the SPIO magnetization saturates to 600 mT. By comparison, the nuclear paramagnetism of water in a 7 T MRI scanner is only 27 nT. That is, MPI images a magnetization that is 22 million times more intense than we image routinely in high-field MRI. Moreover, biological tissue does not generate or attenuate the low-frequency magnetic fields used in MPI (10 kHz to 1 MHz)^14^, giving the technique ideal contrast independent of source depth. The combination of these characteristics enables MPI to be uniquely suitable for high-contrast, radiation-free cell tracking *in vivo*^15^.

To test MPI for tracking cells *in vivo*, we built two small animal MPI scanners, the construction of which are previously described and analyzed^16^,^17^. These include a projection-format FFL scanner with 2.35 × 2.35 T/m gradient, which achieves roughly 4 mm × 8 mm resolution along and orthogonal to the axis of the imaging bore respectively using undiluted Resovist tracer^18^. The FFL scanner’s projection-mode image acquisition enables fast scanning times on the order of seconds for a small animal-sized imaging volume. We also report the use of a field-free point (FFP) MPI scanner with a 7 × 3.5 × 3.5 T/m gradient, which enables roughly 2 mm × 4 mm × 2 mm resolution using Resovist and higher resolution using MPI-tailored SPIOs^17^,^19^. An example MPI image of labeled cells scanned using the FFP scanner is shown in Fig. 2. Both scanners use our group’s recent advances in MPI reconstruction that are necessary and sufficient for linear quantification^13^. We also note that the experimental detection sensitivity of the FFL scanner is, at roughly 5 ng Fe/voxel, 15-fold better than the FFP scanner, as expected due to a larger voxel volume. Based on the high sensitivity and faster scan times of the FFL scanner, we conducted all MPI imaging in this study, with the exception of the image shown in Fig. 2, using the FFL scanner.

## Results

### Linear and Absolute Quantification

**Figure 1:**
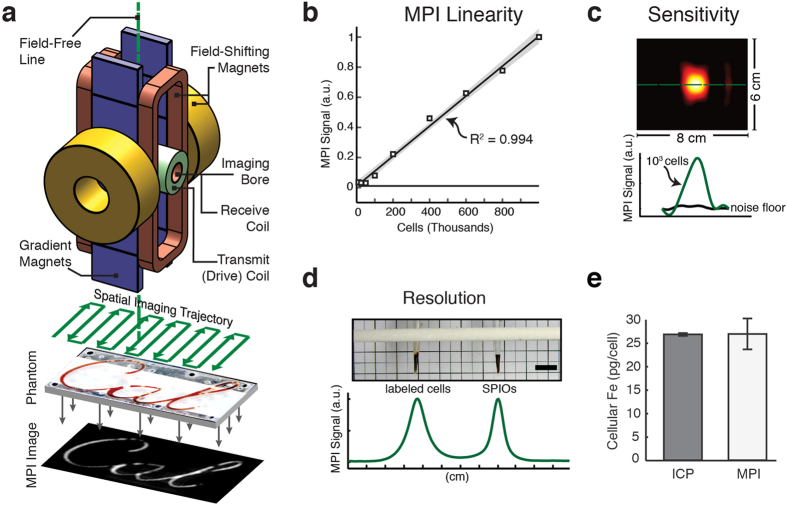
MPI is a tracer imaging modality that can image tagged cells using time varying magnetic fields. (**a**) Diagram of a projection MPI scanner. Three superposed magnets (field-shifting magnets, gradient magnets, and transmit coil) produce and translate a magnetic field-free line (FFL) across an imaging volume. As the FFL is rastered across a distribution of superparamagnetic iron oxide (SPIO) particles using a spatial imaging trajectory, the particle ensemble magnetization changes in magnitude and orientation in response. The changing particle magnetization is detected via a detector coil and reconstructed to form an image. (**b**) Plot of MPI signal from 9 SPIO-labeled cell populations ranging from 1 × 10^4^ to 1 × 10^6^ cells, shown with MPI system noise floor. SPIO signal in MPI is linear with cell number (R^2^ = 0.994, linear fit and 95% confidence bounds for fitted slope shown). (**c**) Detection threshold for MPI cell tracking in FFL scanner. Approximately 1,000 SPIO-labeled hESC-derived cells in a 100 μL *in vitro* suspension were imaged using MPI and compared to an empty scan. The signal-noise ratio of the image is estimated at above 5, giving a detection limit of approximately 200 cells. This represents the current detection sensitivity limit of our FFL MPI scanner, but the theoretical detection limit may be as low as a single cell. FOV: 6 cm × 8 cm. 20 second MPI acquisition. (**d**) MPI 1D line profile of Resovist-labeled cells and Resovist point sources. The FWHM resolution of the MPI signal is approximately 1.5-fold better for Resovist SPIO particles alone, at 5 mm, than for SPIO particles transfected into cells, at 7.7 mm. Scale bar: 1 cm. (**e**) MPI estimates for average cellular iron content (27.0 ± 3.3 pg/cell) correspond with ICP analysis (26.8 ± 0.3 pg/cell), making MPI useful for nondestructive iron quantification. n = 3 for ICP and n = 4 for MPI; results are mean ± SD.

We first asked whether our MPI scanners could quantify SPIO-labeled cells *in vitro*. We modified an established MRI labeling protocol to label hESC-derived cells with Resovist SPIO tracer using a protamine sulfate transfection agent^5^. Both Resovist and protamine sulfate are approved for clinical use in the European Union and have no significant effect on cell proliferation, viability, and differentiation^5^,^6^,^7^. Using the projection-mode MPI imager, we imaged nine labeled hESC-derived cell populations ranging from 1 × 10^4^ to 1 × 10^6^ cells. The normalized MPI signal was linear to the number of cells imaged (R^2^ > 0.994, Fig. 1b). In equivalent control cell populations without iron oxide labeling, no MPI signal was detected.

We further asked if MPI allows absolute quantification of iron oxide tracer. To determine this, we created a calibration curve using the MPI signal from measured volumes at 0, 0.3, and 1 μL of Resovist SPIO tracer. We then used the calibration curve to estimate average cellular SPIO content in MPI images of 1 × 10^6^ labeled cells. These MPI cellular iron estimates, at 27.0 ± 3.3 pg/cell, were within 99% agreement (P = 0.923) with standard but destructive Inductively Coupled Plasma (ICP) measurements at 26.8 ± 0.3 pg Fe/cell (Fig. 1e).

### Sensitivity

We next investigated the sensitivity threshold of the prototype FFL MPI scanner for imaging labeled cells. We imaged 1 × 10^3^ *in vitro* tagged cells in a pellet using a 20 second projection MPI scan and reconstructed the final MPI image using only the second and third harmonics of the drive field frequency. The reconstructed image showed a signal-noise ratio (SNR) greater than 5, corresponding to a 200-cell (5.4 ng Fe) detection limit (Fig. 1c). This detection limit corresponds to 130 nM sensitivity for a measured voxel volume of 750 μL.

### Resolution

To assess the effect of SPIO cell labeling on MPI resolution, we acquired and compared a MPI line scan of two point sources: (a) 8 × 10^6^ labeled hESC-derived cells and (b) 20 μL of 10× diluted Resovist tracer (Fig. 1d). The point sources were placed 3.5 cm apart on a 3D printed sample holder. The point sources were small (1.3 mm width) compared to the native resolution of the FFL system. The axial dimension of the point sources did not affect resolution analysis because the axis of each vial was placed parallel to the FFL during imaging. After a native MPI image was acquired, a line profile was generated across the center of the sample image to assess image blurring (Fig. 1d, bottom panel). A comparison of the MPI signal widths of the two samples showed that the full-width-at-half-maximum (FWHM) resolution of diluted Resovist particles in solution was 5 mm, approximately 1.5-fold better than that of the labeled cells at 7.7 mm. The measured native resolution for Resovist at 5 mm matches well with theory^11^, and approaches for improving the resolution are explored in Discussion.

### Longitudinal Cell Tracking

Last, we tested MPI for tracking neural progenitor cells (NPCs) *in vivo*, which have previously shown promise as treatments for Parkinson’s Disease, Huntington’s disease, epilepsy and ischemia^20^. It is known that NPCs survive, migrate, and show reconstructive properties when implanted in rodent models of forebrain ischemia^21^,^22^. The high specificity of MPI for SPIO imaging would facilitate quantification of cell delivery and retention, leading to better understanding of dynamic events such as graft movement in such brain disease models.

To evaluate MPI for long-term tracking of neural implants, we stereotactically implanted 5 × 10^5^ SPIO-tagged hESC-derived NPCs in 5 μL PBS solution in the forebrain of two immunosuppressed rats, representing a standard therapeutic dose for neural cell implants^21^,^22^,^23^. To assess MPI monitoring of cell graft mobility and clearance *in vivo*, we also implanted an equivalent number of labeled NPCs near the lateral ventricle of a third animal to facilitate translocation and potential clearance of the graft. 0.15 μL Resovist tracer diluted in 4.85 μL PBS was administered to the forebrain of a fourth animal as a control. Due to institutional policy, we began longitudinal imaging of the animals post-recovery at day 10 for animals 1–3 and day 4 for animal 4. We then monitored the graft signals using MPI imaging for a period of 87 days post-administration. Serial MPI images of cell grafts show high signal contrast with no detectable signal from surrounding anatomy (Fig. 3a). Figure 3b shows total MPI iron signal from the cell graft in animals 1-2 had non-significant decay over time. In contrast, the cell graft in animal 3 exhibited the presence of iron caudal/posterior to the implant site and significant iron clearance compared to animals 1-2, suggesting movement and clearance of NPCs throughout the ventricle. The control animal receiving SPIO tracer alone showed no long-term detectable MPI signal.

### Histological and MRI Validation

We then compared MPI cell tracking data to postmortem Prussian blue (PB) staining and MRI (Fig. 4). Animals 1 and 2 (Fig. 4a) showed the presence of SPIOs only at the injection site. In Animal 3, PB staining and MRI also revealed the presence of SPIOs adjacent to the lateral ventricle (Fig. 4c), which confirmed the MPI observation that tagged cells had migrated posteriorly throughout the ventricle. Additional postmortem PB staining did not show any residual iron in the brain of the animal that received tracer alone, indicating rapid tracer clearance seen in MPI images. Immunohistochemical staining against neural progenitor markers nestin, and human-specific neural cell adhesion molecule (NCAM), and human cytoplasmic marker SC121 confirmed the presence of human SPIO-labeled cells at the administration site (Fig. 4b). Expression of the macrophage/microglial marker CD68 showed some immune cell infiltration within the grafts. CD68 staining also indicated the presence of iron-containing immune cells along the lateral ventricle in Animal 3, indicating immunogenicity as a mechanism of graft clearance.

## Discussion

In this study, we describe the use of longitudinal *in vivo* MPI to track and quantify implanted neural cell grafts over three months. We also demonstrate that the MPI signal is linear and can be used to quantify cell number *in vivo*, and that our projection format MPI scanner has a detection sensitivity of as few as 200 cells *in vitro*. As a comparison, MRI, SPECT, PET, and fluorescent imaging are reported to have robust detection limits greater than 10^4^ cells, with only bioluminescent imaging able to detect 10^3^ cells superficially implanted in small animals^3^. We note that in the described prototype MPI scanners, noise in the detector coil preamplifier, at around 1 nV/√Hz, is not optimized and can be further improved by 1-2 orders of magnitude. This preamplifier noise exceeds unwanted harmonic interference and is currently the dominant noise source in our MPI systems. Thus, with further instrumentation development, the true physical limit of *in vivo* detection for MPI may range between 1–10 cells at scanning times between seconds to minutes.

An area of promising research in MPI is improving the spatial resolution, now roughly 1 mm with a 7 T/m magnetic gradient. Here, we employed 2.35 T/m gradients for a FFL scanner, which yielded approximately 7 mm spatial resolution when imaging cells labeled with Resovist tracer. Spatial resolution in MPI is predicted to increase linearly with the gradient field strength and cubically with improved nanoparticles^11^. For example, previously published 22 nm particles enable double the image resolution of Resovist^19^, which behaves similar to a solid core 17 nm particle^18^. Many groups continue to develop MPI-optimized contrast agents for improved MPI resolution^24^,^25^,^26^,^27^,^28^.

We routinely observed an intriguing change in MPI spatial resolution between Resovist tracer alone and Resovist-labeled cells, an effect also noted in previous studies on the MPI signal of SPIO-labeled red-blood cells^29^,^30^. Possible mechanisms for this resolution change could include size-selective endocytosis of transfection agent-Fe complexes^5^,^31^, increased MPI relaxation effects due to increased intracellular viscosity^32^,^33^, and transfection agent- mediated nanoparticle aggregation that may cause changes to the bulk particle magnetization and relaxation properties^34^,^35^,^36^. A better understanding of the mechanism underlying this phenomenon would not only enable tailored nanoparticles for MPI cell tracking, but could also potentially be exploited to generate image contrast based on nanoparticle relaxation or clustering. For example, changes in particle relaxation due to changes in pH or viscosity may be used to differentiate between different physiological conditions in live and dead cell populations, detect cell apoptosis, or image *in vivo* uptake of SPIOs by immune cells.

It is clear that the direct detection of SPIO labels in MPI can enable studies that require *longitudinal* monitoring of cells in anatomy previously inaccessible to optical, MRI, and nuclear techniques, such as the GI tract, pulmonary vasculature, and near bone. MPI’s high image specificity for SPIOs enables visualizing systemic cell retention, migration, and biodistribution post-administration^3^. These systemic monitoring applications could include non-invasive probing of the immune system, such as the monitoring of labeled macrophages migrating to sites of inflammation^3^,^37^,^38^ and monitoring the fate of labeled T-cells used in immunotherapies. Finally, the SPIO labels and magnetic fields used in MPI have been shown to be human-safe^17^, enabling a clear path to clinical MPI translation.

## Methods

### Magnetic labeling of differentiated human embryonic stem cells (hESCs) and determination of labeling efficiency

H1 hESCs (WiCell, Madison, WI) were cultured on Matrigel-coated cell culture plates (BD, Franklin Lakes, NJ) in *X-Vivo* medium (Lonza, Norwalk, CT) supplemented with 80 ng/mL FGF- 2 (PeproTech, Rocky Hill, NJ) and 0.5 ng/mL TGF-β1 (R&D Systems, Minneapolis, MN), as described previously^39^.

For embryoid body (EB) mediated differentiation, H1 colonies were enzymatically isolated from Matrigel-coated plates by 5-minute treatment with 1 mg/mL collagenase type IV (Worthington Biochemical Corporation, Lakewood, NJ) and partially dissociated by gentle pipetting. The resulting cell clusters were resuspended in hESC culture medium, *X-Vivo* without FGF-2 and TGF-β1, and transferred to ultra-low-attachment plates (Corning Incorporated, NY) for cell aggregation for 4 days. The generated EBs were then seeded on Matrigel-coated plates and allowed to differentiate for an additional 10–12 days in adherent conditions in *X-Vivo* medium.

For cell labeling, Resovist superparamagnetic iron oxide particles (Bayer Schering Pharma AG, Leverkusen, Germany; 0.5 mmol Fe/mL) at a concentration of 200 μg Fe/mL were incubated with 6 μg/mL protamine sulfate (American Pharmaceuticals Partner, Schaumburg, IL) in serum free *X-Vivo* medium (Lonza, Walkersville, MD) and allowed to form complexes on a shaker for 6 hr. The solution containing Fe-Pro complexes was added to differentiating hESCs in adherent culture condition. The cultures were incubated with Fe-Pro complexes overnight. Cultures after labeling were washed with 10 U/mL heparin (Sigma) in PBS three times to dissolve remaining surface-bound Fe-Pro complexes.

For assessment of labeling efficiency, Prussian blue cellular staining was performed by incubating cells, fixed with 4% paraformaldehyde, in a mixture of 4% potassium ferrocyanide and 3.7% HCl (Iron Stain Kit, Sigma) for 20 minutes to visualize intracellular iron particles. Cells were counted manually under a microscope to determine average cell labeling efficiency. Labeling efficiency was found to be 71% +/− 8%. A hemocytometer was used to determine all cell numbers.

### MPI image acquisition

**Figure 2:**
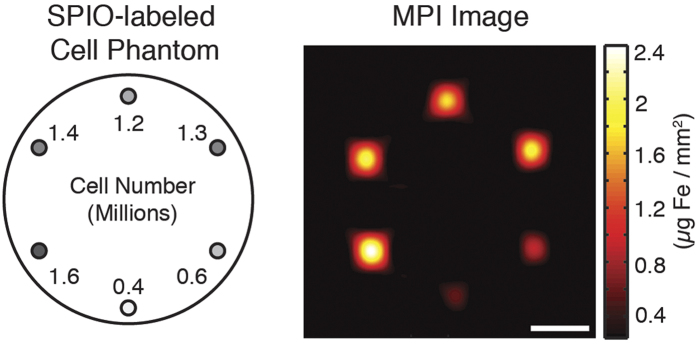
FFP imaging of an acrylic phantom filled with six populations of SPIO-labeled cells. The MPI signal from cell populations (ranging from 4 × 10^5^ to 1.6 × 10^6^ in number) corresponds linearly to iron oxide tracer concentration, enabling quantitative imaging. Imaging parameters: 3.5 min acquisition on a 7 T/m 3D MPI scanner, 5 cm × 4.5 cm × 3 cm FOV. Scale bar: 1 cm.

**Figure 3:**
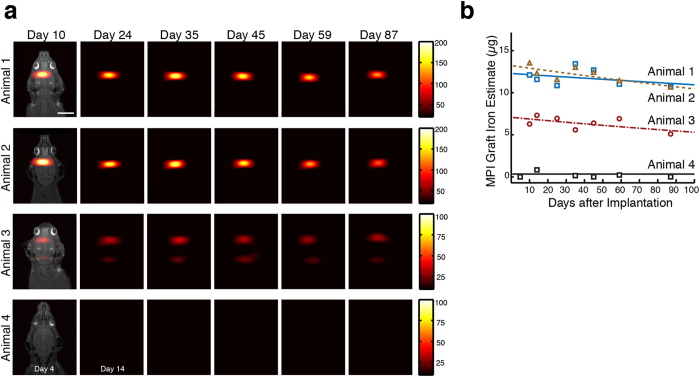
MPI quantitatively tracks NPC neural implants in rats over 87 days. (**a**) Longitudinal MPI imaging of 5 × 10^5^ SPIO-labeled human NPCs implanted in the forebrain cortex (Animals 1-2), near lateral ventricle (Animal 3), and equivalent SPIO-only tracer in the forebrain cortex as control (Animal 4). MPI imaging quantifies graft clearance and movement over time, with rapid total clearance of SPIO-only injection (MPI: 30 sec acquisitions, 9.3 cm × 6 cm FOV, color intensity in ng/mm^2^, MRI reference: 10 min acquisitions, 7.5 cm × 4 cm, 384 × 384 matrix). All images are equally scaled; scale bar: 1 cm. (**b**) Total iron MPI estimates for *in vivo* cell grafts are plotted as a function of time with exponential fit. *In vivo* iron in Animal 1 and 2 do not show significant decrease over time, while iron signal was significantly decreased in Animal 3 starting Day 10. Animal 4 showed no long-term persistent MPI signal.

**Figure 4:**
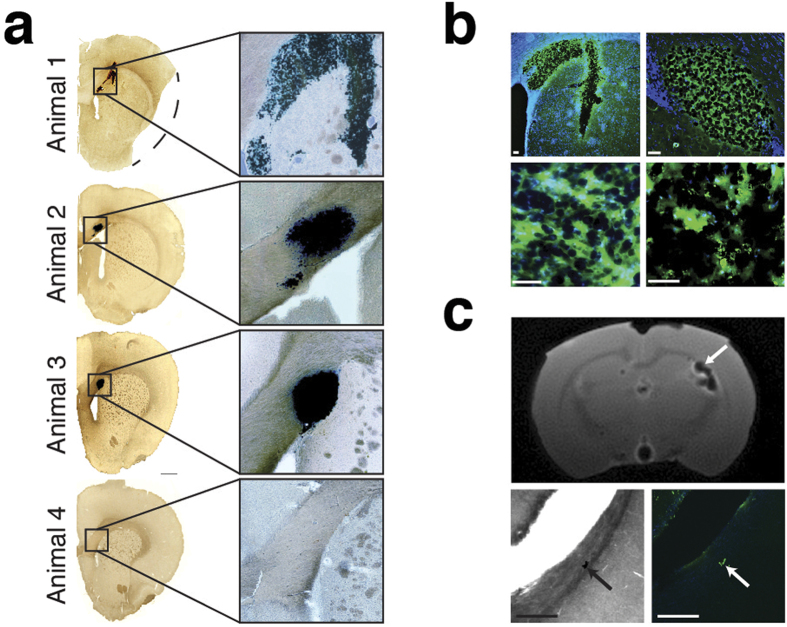
Histological and MRI validation of iron location and quantification. (**a**) Histological analysis of NPC grafts. Postmortem Prussian blue (PB) staining confirms presence of iron-labeled cells at administration site but not for SPIO-only control (Animal 4). (**b**) Representative immunohistochemical slices (top right panels) are shown for NPC marker nestin (top left, Animal 1), neural cell adhesion molecule (top right, Animal 3), and human-specific cytoplasmic marker SC121 (bottom left, Animal 2), which indicate iron label within NPC grafts. CD68 staining (bottom right, Animal 3) also indicates immune cells at administration site, suggesting immune-based graft clearance. Scale bars: 100 μm. (**c**) Postmortem axial MRI indicates iron in lateral ventricle in Animal 3 (arrow). PB and CD68 staining of lateral ventricle in Animal 3 shows SPIO uptake by immune cells. MRI: 20 min acquisition, 2.56 cm isotropic FOV, 256^3^ pixel matrix. Scale bars: 1 mm.

Two custom-built MPI systems were used in this study: 1) a projection MPI scanner with 2.35 T/m/μ0 field gradient^18^, and 2) a 3D MPI scanner with 7 T/m/μ0. Both systems used a drive field frequency of 20.05 kHz and excitation strength of 40 mTpp/μ0, with 30 second total scan time for projection MPI scanner and 3 minute scan time for 3D MPI scanner unless otherwise specified. MPI images were reconstructed using an x-space MPI reconstruction^17^,^38^,^12^, followed by light Wiener deconvolution using a measured point-spread function. All MPI images were windowed at 10% full scale. All data acquisition, control, and data processing were performed with MATLAB (Mathworks). All MPI imaging was conducted using the projection FFL scanner with the exception of the cell phantom imaging study shown in Fig. 2.

### Determination of linearity of MPI signal with cell number

To investigate the linearity of the MPI signal with imaged cell number, we imaged 9 populations of Resovist-labeled hESC-derived cell pellets containing 10 × 10^3^, 25 × 10^3^, 50 × 10^3^, 100 × 10^3^, 200 × 10^3^, 400 × 10^3^, 600 × 10^3^, 800 × 10^3^, and 1,000 × 10^3^ cells using a projection-format MPI scanner as described above. Following image reconstruction, the maximum signal intensity for each MPI image was determined and the Pearson correlation coefficient was determined between normalized MPI signal and imaged cell number. Subsequent linear regression on the data and 95% confidence intervals for the slope of the regression line were determined.

### Intracellular Iron Measurement

Average intracellular iron was determined using induction-coupled plasma optical emission spectrometry (Optima 5300 DV, PerkinElmer, Waltham, MA) on a population of 1 × 10^6^ SPIO-labeled hESC-derived cells. Prior to measurement, cells suspended in 0.2 mL PBS solution were digested using a 1.8 mL mixture of 70% nitric acid and 30% hydrogen peroxide and diluted to 60 mL using deionized water. ICP analysis was performed in triplicate to determine intracellular iron as mean ± SD in picograms of iron per cell. Intracellular iron was also measured using MPI. A calibration curve for volume MPI signal per picogram iron was first generated using 0, 0.3, and 1.0 μL Resovist tracer. Subsequently, we performed MPI imaging of 1 × 10^6^ labeled cells (N = 4 each) and determined intracellular iron using the calibration curve as mean ± SD in picograms of iron per cell. To evaluate the congruence between MPI and ICP intracellular measurements, we performed an unpaired two-sample T-test for mean intracellular iron, with significance level set to 0.05.

### *In vivo* stem cell implantation and MPI imaging

All animal procedures were conducted according to the National Research Council’s Guide for the Care and Use of Laboratory Animals and approved by UC Berkeley’s Animal Care and Use Committee. Immunocompetent 8-week old female Fischer 344 rats weighing 145 g were used for *in vivo* imaging. All animals were fed on an ad libitum diet of Teklad Rodent Diet 2018 (Harlan, Indianapolis, IL).

For *in vivo* MPI cell tracking experiments, hESCs were differentiated to neural progenitor cell by dual inhibition of SMAD signaling^40^ using SB431542 (10 μM) and LDN-193189 (1 μM) in adherent conditions for 12 days. The generated NPCs were labeled with Resovist SPIO particles as previously described here. The cell suspension contained approximately 100 × 10^3^ viable cells/μL.

Animals were anesthetized with an intraperitoneal injection of ketamine (100 mg/kg rat weight) and xylazine (7.5 mg/kg), positioned into a stereotactic frame. For two animals, a total of 500 × 10^3^ SPIO-labeled cells in 5 μL were injected at the following stereotaxic coordinates: 1.0 mm AP (relative to Bregma), 2.0 mm lateral, 2.5 mm ventral (from dura) over 10 min using a Hamilton syringe at a rate of 0.5 μL/min. For Animal 3, the coordinates were adjusted to 1.0 mm AP (relative to Bregma), 1.5 mm lateral, and 3.5 mm ventral (from dura). A control animal was injected using 0.15 μL of Resovist tracer diluted in 4.85 μL PBS using the same stereotactic coordinates as Animals 1-2. The needle was left in place for 3 min after injection and slowly removed. Animals were allowed to recover from surgery for up to ten days before MPI imaging, with 7 daily injections of cyclosporine-A starting 1 day prior to implantation at a dose of 15 mg/kg body weight.

Animals were imaged using MPI at days 10, 24, 35, 45, 59, and 87 post-implantation. Animal 4 was imaged at days 4 and 14 instead of days 10 and 24 with all other time points remaining the same. During *in vivo* MPI imaging, animals were induced into anesthesia using 2.5–3.0% isoflurane and maintained at 1.5% isoflurane at 2 L/min and placed in a custom-built animal bed in the MPI scanner. *In vivo* MPI scans were conducted on the projection MPI scanner using a 9.3 cm by 6 cm FOV with 30 second scan time.

For determination of *in vivo* SPIO clearance rates measured by MPI, a linear regression was performed on the logarithm of the time-varying volumetric MPI signal for each animal.

### Postmortem MRI

*Ex vivo* brains were imaged on a 7T Bruker PharmaScan MRI scanner using a ZTE pulse sequence. MR scans had total acquisition times of 20 minutes with 256^3^ voxels and 100 μm^3^ voxel size at 2.9 degree flip angles and 4 ms T_R_. Anatomical MRI images were acquired with postmortem age-matched animals using a TurboRare pulse sequence with 10 minute acquisition and 384^2^ voxels, 7.5 cm × 4 cm FOV, and 2.5 sec T_R_.

### Stereology and Immunohistological Analysis

The brains were fixed in 4% paraformaldehyde overnight, then cryopreserved in 20% sucrose, frozen in dry ice, and cut in 40 μm coronal sections using a microtome. For immunohistochemistry, the following primary antibodies were used: mouse anti-nestin (1:500, R&D system), SC121 (1:400, Stem Cells Inc.), mouse anti-CD68 (1:100, Abcam). Sections were then incubated with fluorescent-labeled secondary antibodies Alexa 488 (green) or Alexa 568 (red)-labeled goat IgG; 1:1000, (Life Technologies) for 2 hr at room temperature and then mounted on glass slides. Brain slides were counter-stained with DAPI and imaged using a Zeiss Axio Observer A1 inverted microscope or a confocal microscope (LSM 710, Zeiss).

## Additional Information

**How to cite this article**: Zheng, B. *et al.* Magnetic Particle Imaging tracks the long-term fate of *in vivo* neural cell implants with high image contrast. *Sci. Rep.* **5**, 14055; doi: 10.1038/srep14055 (2015).
